# Graphene Oxide@Heavy Metal Ions (GO@M) Complex Simulated Waste as an Efficient Adsorbent for Removal of Cationic Methylene Blue Dye from Contaminated Water

**DOI:** 10.3390/ma15103657

**Published:** 2022-05-20

**Authors:** Yangfan Ding, Zhe Chen, Jinglei Wu, Ahmed I. Abd-Elhamid, Hisham F. Aly, AbdElAziz A. Nayl, Stefan Bräse

**Affiliations:** 1 Key Laboratory of Science and Technology, Eco-Textile & Shanghai Engineering Research Center of Nano-Biomaterials and Regenerative Medicine, College of Chemistry, Chemical Engineering and Bio-Technology, Donghua University, Shanghai 201620, China; dingyangfan@mail.dhu.edu.cn (Y.D.); chenzhe@mail.dhu.edu.cn (Z.C.); jw@dhu.edu.cn (J.W.); 2 Composites and Nanostructured Materials Research Department, Advanced Technology and New Materials Research Institute, City of Scientific Research and Technological Applications (SRTA-City), New Borg Al-Arab 21934, Egypt; ahm_ch_ibr@yahoo.com; 3 Hot Laboratories Center, Egyptian Atomic Energy Authority, Cairo 13759, Egypt; alydrhisham@yahoo.com; 4 Department of Chemistry, College of Science, Jouf University, Sakaka 72341, Al Jouf, Saudi Arabia; 5 Institute of Organic Chemistry (IOC), Karlsruhe Institute of Technology (KIT), Fritz-Haber-Weg 6, 76133 Karlsruhe, Germany; 6 Institute of Biological and Chemical Systems-Functional Molecular Systems (IBCS-FMS), Director Hermann-von-Helmholtz-Platz 1, 76344 Eggenstein-Leopoldshafen, Germany

**Keywords:** graphene oxide, heavy metal ions, nanocomposite, cationic dye, adsorption, water treatment

## Abstract

Graphene oxide (GO) was heavily used in the adsorption process of various heavy metal ions (such as copper (Cu) and iron (Fe) ions), resulting in a huge waste quantity of graphene oxide@metal ions complex. In this research, the authors try to solve this issue. Herein, the GO surface was loaded with divalent (Cu^2+^) and trivalent (Fe^3+^) heavy metal ions as a simulated waste of the heavy metal in various removal processes to form GO@Cu and (GO@Fe) composites, respectively. After that, the previous nanocomposites were used to remove cationic methylene blue (MB) dye. The prepared composites were characterized with a scanning electron microscope (SEM), transition electron microscope (TEM), Fourier transmission infrared (FTIR), Raman, and energy-dispersive X-ray (EDS) before and after the adsorption process. Various adsorption factors of the two composites towards MB-dye were investigated. Based on the adsorption isotherm information, the adsorption process of MB-dye is highly fitted with the Langmuir model with maximum capacities (mg g^−1^) (384.62, GO@Cu) and (217.39, GO@Fe). According to the thermodynamic analysis, the adsorption reaction of MB-species over the GO@Cu is exothermic and, in the case of GO@Fe, is endothermic. Moreover, the two composites presented excellent selectivity of adsorption of the MB-dye from the MB/MO mixture

## 1. Introduction

Recently, synthetic dyes are more highly required in numerous industries than natural dyes; this is attributed to not being expensive, easy to fabricate, high stability, and having a wide color range [1]. With increasing the reliance of different industrial applications on synthetic dyes, the water pollution problem from the industrial discharge of contaminated dye increases. These effluents produced from the various industries without any suitable purification of the raw water cause a negative effect on ecosystems by reducing the availability of sunlight. So, there is an urgent requirement for efficient industrial wastewater treatment.

Methylene blue (MB) is a cationic dye widely applied in several applications such as textile, paper, pigments, and pharmaceutical industries. It reduces the rates of photosynthesis processes by preventing light penetration and dissolved oxygen levels in the entire ecosystem. MB-dye has harmful effects on humans and the entire aquatic biota, where it causes vomiting, shortness of breath, high blood pressure, headache, hypertension, and mental confusion [2]. In addition, their higher thermal, photo stabilities, and biodegradation resistance have dangerous effects on the entire aquatic biota. Therefore, the industrial discharge containing methylene blue dye must be managed before draining into the raw water.

Nemours techniques were employed for remediation of MB-dye from an aqueous solution, such as; Photocatalytic degradation [3], catalytic reduction [4], catalytic degradation [5], ozonation [6], biodegradation [7], electrocoagulation [8] and adsorption [9]. Adsorption methods are widely utilized for treating contaminated water due to their cost-effect, ease of setting up, flexibility, simplicity, and high efficiency.

During the last decades, graphene oxide (GO) and its modification forms were investigated, promising adsorbents due to their unique properties. GO is a 2D carbonaceous nanomaterial with a thickness of one C-atom derived from graphite’s strong oxidation. The oxidation step will be generated enhanced densities of various oxygen functional groups on the surface of GO in the forms of (–OH, C–O–C, C=O) on the GO-sheet basal plane and (–COOH) located at the edge. These O-functional groups will enhance the hydrophilicity of the GO nanosheets, which provide good contact with the aqueous solution. In addition, the containing oxygen atoms have lone pair electrons. Therefore, by sharing these electron pairs, they can suitably bind with the cationic species. Moreover, the 2D structure awards the GO large surface area that enhances its adsorption capacity and reduces adsorption time. Therefore, GO has extensive used in removal of different heavy metals from aqueous media such as copper (Cu) [10,11], lead (Pb) [12], cobalt (Co) [13], iron (Fe) [14] cadmium (Cd) [15].

However, the adsorption of heavy metal ions using GO nanosheets occurs via strong surface interactions between the oxygen-functional groups on the GO surfaces and metal ions [16]. Therefore, the adsorbent GO regeneration will require aggressive conditions. For example, Wang et al. [12] treated the GO-Pb complex with 1.0 M HNO_3_ to desorb the Pb (II) ions from the surface of GOs and then washed it with Milli-Q water. Wang et al. [13] recovered the GO from GO–Co (II) using 1 mol/L Na_2_CO_3_, and the desorption process proceeded for 48 h and was rinsed with Milli-Q water several times. Pakulski et al. [15] observed the blocking of active sites of the GO using of 0.1 M solution of ethylenediaminetetraacetic acid (EDTA) to regenerate the GO from GO–Pb(II), GO–Cd(II), and GO–Cu(II), which will cause reducing in the adsorption efficiency. Wu et al. [17] desorbed 74% of Cu (II) from Cu (II) capture GO using 25 mL of HCl solution at pH = 1, and the desorption time reached 2 h. In most previous cases, the regeneration process appears to be not straightforward and increases the cost-effect of the whole operation, where it requires high acidic media, washing several times with distilled water, long desorption time, and low reusing efficiency.

Therefore, this work investigates the use of the GO-heavy metal (GO@M) complex that yielded from the treating the heavy metals aqueous solution in secondary removal of the dye species from polluted water. In this work, Cu (II) and Fe (III) as a model of divalent and trivalent heavy metal ions were selected to be loaded over the GO surface to produce CO@Cu and GO@Fe composites. These graphene oxide@metal ion (GO@M) complexes acted as a simulated solid waste produced from heavy metal removal experiments and were reused to adsorb MB-dye from the aqueous media. Both used composites GO@Cu and GO@Fe were successfully characterized before and after removing MB-dye species using various advanced techniques. Different adsorption parameters and selectivity of adsorption of the cationic MB dye were studied.

## 2. Materials and Methods

The purity grade of all used chemicals and more detailed information about instruments used in this work was explained in the Supplementary Material File.

### 2.1. Preparation of GO

GO was prepared as reported in our previous research [9]. Briefly, 3.0 g of graphite powder was introduced into a mixture of an acid solution of (110 mL concentrated H_2_SO_4_ + 15 concentrated H_3_PO_4_) and stirred for 10 min. Subsequently, 9.0 g KMnO_4_ was added gradually over 1.0 h, and the temperature kept <20 °C. After that, 2.0 g of NaNO_2_ were added gradually to complete the oxidation process and kept stirring for 24 h. the obtained brown suspension was added to 500 mL H_2_O and stirred for 1.0 h, followed by the addition of 15 mL H_2_O_2_ to produce a yellow suspension. The solid sample was collected by filtration and washed with distilled water. The GO suspension was then dispersed in distilled water at a 3.0 mg mL^−1^ concentration and stored for further use.

### 2.2. Preparation of GO@Cu or GO@Fe Composites

To prepare the GO@-Cu or GO@Fe composites, 3.0 g of Cu (NO_3_)_2_·3H_2_O or FeCl_3_ was dissolved in 800 mL distilled water. After that, 50 mL (3.0 mg mL^−1^) GO suspension was added to the previous metal solution and kept stirring at 50 °C for 24 h. The yielded composites GO@Cu or GO@Fe were separated by filtration, washed several times with distilled water, dried at 70 °C, and stored for further use.

### 2.3. Batch Adsorption of MB-Dye

MB-dye stock solution was prepared by adding 0.1 g to 100 mL distilled water and stirring for 24 h for complete dissolution. The induce of stirring time on the removal of MB was tested by introducing 5.0 mg GO@Cu or 6 mg GO@Fe into the dye solution (50.0 mL, 50 mg L^−1^) at neutral pH and room temperature for known time intervals. Initial MB-dye concentrations (30 to 70 mg L^−1^, GO@Cu) or (10 to 50 mg L^−1^, GO@Fe) were employed to investigate the adsorption isotherm. Definite amounts of the composite (3.0–15 mg, for both composites) were inserted into (50 mL of the aqueous dye solution, GO@Cu) or (30 mg L^−1^, 50 mL, GO@Fe). The pH of the MB-dye solutions was controlled in the range of 1.9 to 11.5 by applying 0.5 mol L^−1^ HCl/NaOH. Salinity experiments were carried out by dissolving known quantities (0.00–0.50 g) of NaCl in the MB-dye solutions. The influence of the temperature on the sorption processes was studied in the range of 25–85 °C. Finally, 2.0 mL of the treated MB-dye solution was isolated, and centrifugation and the dye concentrations were monitored by UV-spectrophotometer at a wavelength of 662 nm. The dye adsorption performance (% R) is expressed as;

% R = ((C_o_ − C_t_)/C_o_) × 100 (1)

where, C_o_ and C_t_ are the initial MB-dye concentrations and the MB-dye concentration at time t, respectively.

## 3. Results and Discussion

### 3.1. Physical and Chemical Characterizations

#### 3.1.1. SEM and TEM

**Figure 1:**
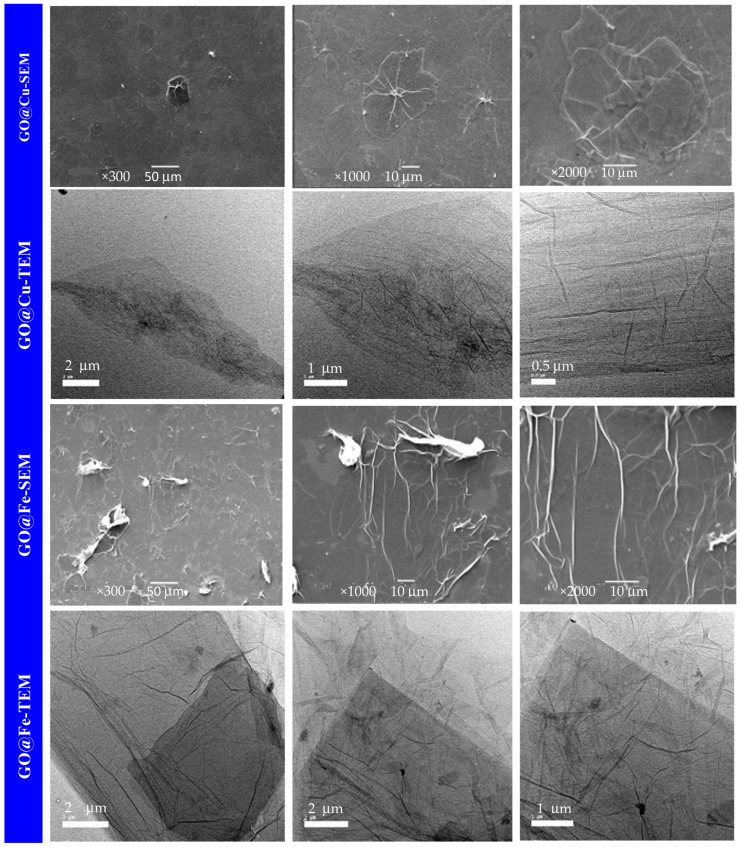

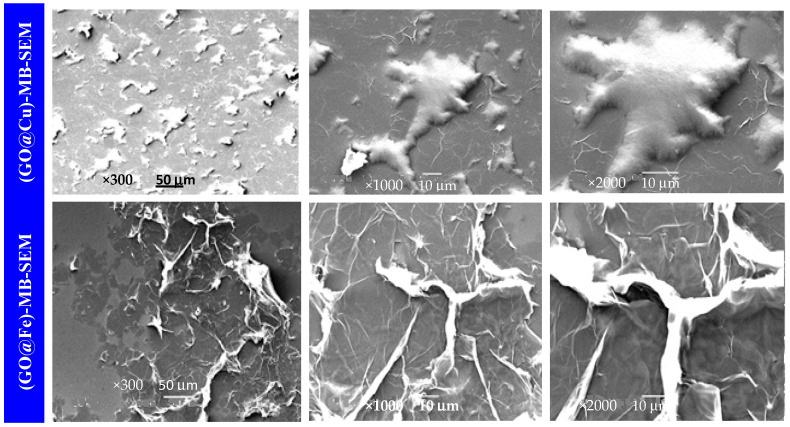
SEM and TEM images for GO@M composite and SEM images for (GO@M)-MB complex at various magnifications (M = Cu^2+^ or Fe^3+^).

The SEM and TEM characterizations were carried out to assess the morphological and structural features of the as-fabricated GO@Cu and GO@Fe samples as represented in Figure 1.

The SEM images showed that the prepared composites at different magnifications exhibit flakes-like structures with smooth surfaces, extended over a large area, and excellent spread over the surface with any noticeable agglomeration. This is also approved by TEM characterization which illustrated in Figure 1. The TEM images at different magnifications presented well quality of the obtained composites, GO@Cu and GO@Fe. Moreover, both samples appear as a single 2D layer with good separation, and no aggregates are found. This structure is highly required in the water treatment application, which maintains almost all the active sites presented on both sides of the layer exposed to the adsorbed species. Consequently, the contact time required for the removal process will reduce, and the adsorbate’s efficiency will be highly enhanced.

Both GO@Cu and GO@Fe samples were characterized using the SEM technique before and after the removal process of the dye at various magnifications and represented in Figure 1. The (GO@Cu)-MB appears at low magnification as dispersed nonuniform constructs; the further magnification showed that the composite layer looks similar to a cloud-like structure, which may be attributed to the complexation between the GO@Cu composite and the MB-dye species. In the case of using GO@Fe, the low magnification demonstrated the presence of a non-ideal layer structure. On the other hand, the high magnification indicated that the composite layer suffers from wrinkles due to interaction with the MB dye.

The TEM images of both GO@Cu and GO-@Fe, which are illustrated also in Figure 1 provides 2D transparent nanosheets. These nanosheets slightly agglomerated loose wrinkles were shown on the surface of the GO@Cu and GO@Fe and can be attributed to the complexation action between the abundant oxygen groups on the GO surface and the M-ion (Cu^2+^ and Fe^3+^). The EDX and elemental mapping corresponding to the TEM images of the GO-@M (M = Cu^2+^ and Fe^3+^) were displayed in Figure 1. 

**Figure 2:**
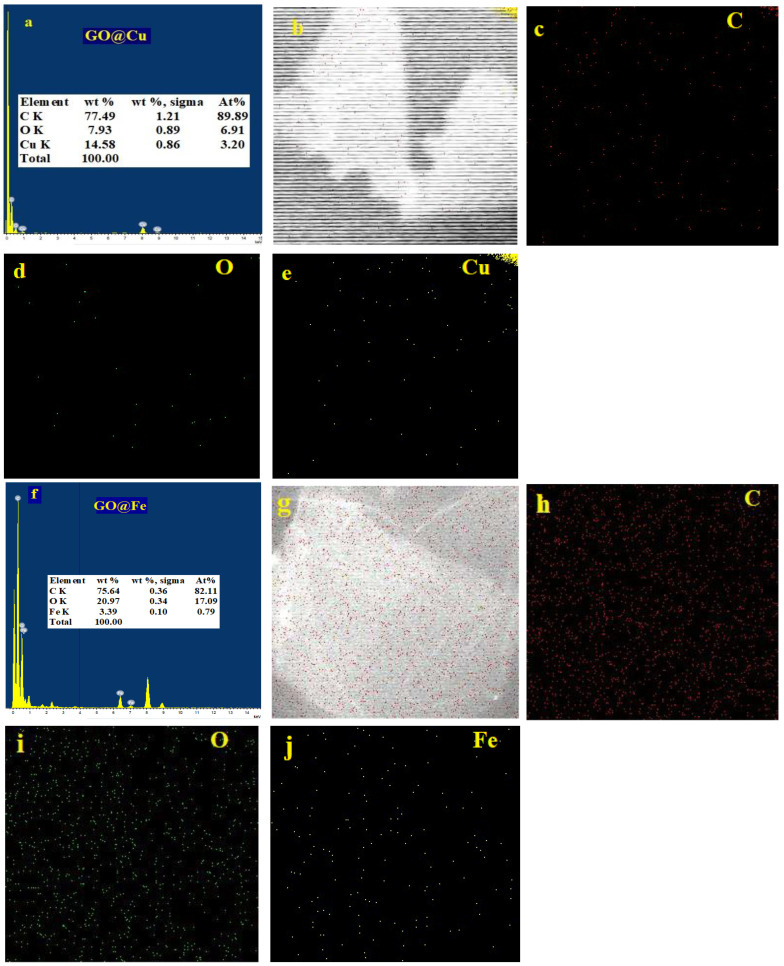
EDX and mapping analysis of GO@Cu (**a**–**e**) and GO@Fe (**f**–**j**).

The EDX results showed that the composites were mainly composed of C, O, and M-ion. Moreover, the mapping analysis provides that both the Cu^2+^ and Fe^3+^ were uniformly distributed over that GO nanosheet surface, as shown in Figure 2.

#### 3.1.2. FTIR

**Figure 3:**
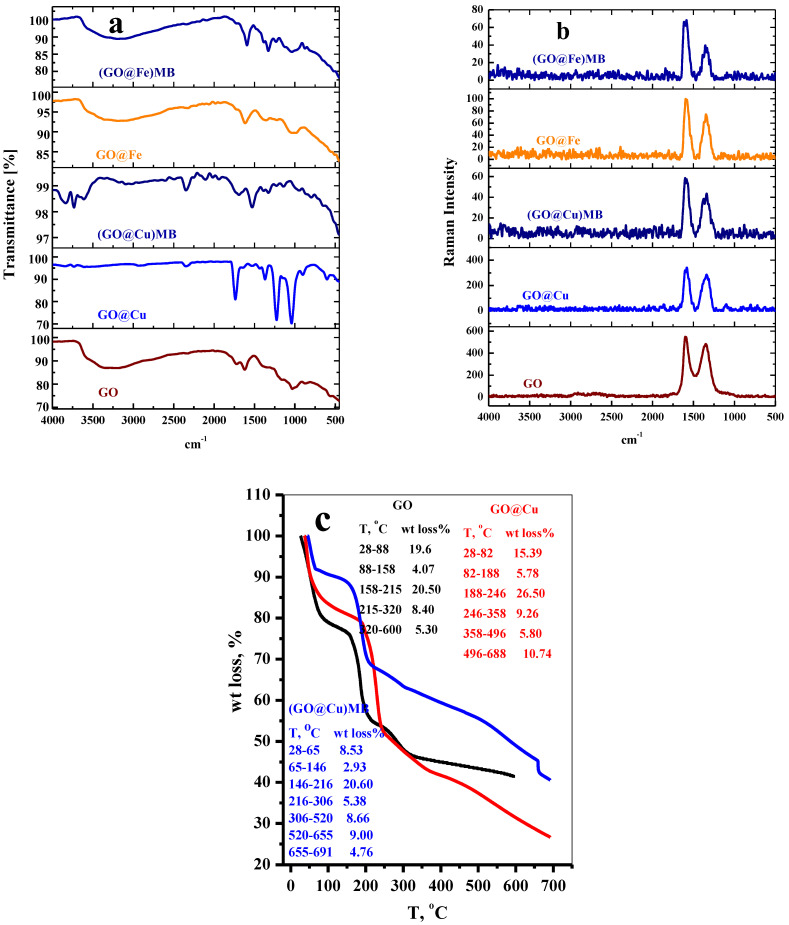
(**a**) FT-IR spectrum, (**b**) Raman spectrum for GO, GO@-M composite and (GO@M)-MB complex (M = Cu^2+^ or Fe^3+^). For GO, GO@M composite and (GO@M)-MB, (M = Cu^2+^ or Fe^3+^), and (**c**) TGA plot for GO, GO@Cu, and (GO@Cu)-MB.

FTIR spectra of the GO, GO@Cu, (GO@Cu)-MB, GO–Fe, and (GO@Fe)-MB were presented in Figure 3a. As indicated, the GO showed its characteristic bands at 3200 cm^−1^ (O–H stretching vibration), 1722 cm^−1^ (C–O stretching vibration), 1622 cm^−1^ (O–H bending vibration), and 1027 cm^−1^ (C–O stretching). The IR spectrum was completely altered upon loading Cu^2+^ over the GO to form the GO@Cu composite, and narrow, sharp bands appeared in the GO@Cu spectrum. In detail, the peaks at 3200 cm^−1^, 1622 cm^−1^, and 1153 cm^−1^ for the GO disappeared, and the peaks at 1722 cm^−1^ and 1027 cm^−1^ (for GO) were shifted to 1736 cm^−1^, and 1041 cm^−1^ (for GO@Cu). New bands at 1369 cm^−1^ (COO–Cu^+^) and 1226 cm^−1^ (C–O–Cu) appeared for the GO@Cu, as shown in Figure 3a. Again, by mixing GO@Cu and the dye species in an aqueous solution, the (GO@Cu)-MB complex resulted in a new different IR spectrum, as shown in Figure 3a. 

Where new bands at 3836–3611 cm^−1^ (attributed to isolated hydroxyl groups) [18], 2494, 1528, and 1140 cm^−1^ (related to the dye species) appeared, and the peaks at 1692 and 1327 cm^−1^ were shifted at 1736 and 1369 cm^−1^, respectively, for GO@Cu. On the other hand, the interaction between the Fe^3+^ and GO for constructing the GO@Fe composite will result in moderated changes in the FTIR spectrum, where the peaks at 3200, 1622, and 1027 cm^−1^ (for GO) will have a shift to 3192, 1614 and 1032 cm^−1^ For (GO@Fe). Furthermore, new peaks at 1224 cm^−1^ (C-O-Fe) and 991 cm^−1^ (O-Fe) appeared in the GO-Fe spectrum. By adsorbing the MB-dye using the GO@Fe composite, a simple shift overall the peaks related to the GO@Fe was obtained. The changes that took place were confirmed. On the one hand, the interactions between the GO and the metal ions (Cu^2+^ & Fe^3+^) and, on the other hand, the successful adsorption of the MB-species on the prepared composites GO@Cu and GO@Fe.

#### 3.1.3. Raman Analysis

The degree of disorder in carbonous materials is evaluated efficiently using the Raman spectroscopy device via the intensity relationships among D and G peaks (I_D_:I_G_). The typical Raman spectra of GO, GO@Cu, (GO@Cu)-MB, GO@Fe, and (GO@Fe)-MB were displayed in Figure 3b. The peaks located at 1352 cm^−1^ (D vibrational band) and 1598 (G vibrational band) are considered the two main characteristic peaks of GO. Where the G peaks are the in-plane vibration peaks of sp^2^ carbon atoms, and the D peaks (sp^3^ hybridization) determine the degree of the disordered due to defects, such as edge defects, vacancies, bond-length, and bond-angle disorder, referring to the I_D_/I_G_ values in Table 1, it was explored that there were small variations between the I_D_/I_G_ value for the GO (1.34) and (GO-@Cu (1.43) and GO@Fe (1.49)) which indicated that the metal ion interacted with the oxygenated function groups without inducing on the C-domain structure. It is important to notice that the I_D_/I_G_ for (GO@Fe)-MB (1.14) highly decreases from GO@Fe (1.49), which may be attributed to the Fe-atom acting as a bridge for the electron transfer from the dye species and the GO and vise-versa.

#### 3.1.4. TGA Analysis

TGA was applied for ascribing the thermal degradation behavior related to the analyzed material and pointed out in Figure 3c. For GO, (showed TGA plot similar to obtained in previous work [9]) a weight loss of approximately 19.6% (28–88 °C) corresponding to evaporation of moisture; 4.06% (88–158 °C) related to interlayer water; 20.50% (158–215 °C) due to degradation of oxygenated function groups; 8.40% (215–320 °C) revealed to dialysis of carboxylic groups. Upon loading of Cu^+^ over the oxygenated function groups of the GO, the GO@Cu composite shows similar thermal decomposition behavior to the pure GO at low temperature. Contrary degradation behavior was observed by increasing the decomposition temperature. In addition, Figure 3c illustrated that the GO@Cu composite possessed high weight loss in the next decomposition stages compared with the pure GO. This behavior may be due to the catalytic action of the Cu at high temperatures, which will enhance the degradation rate of the GO@Cu than GO. By using the GO@Cu as adsorbent for MB-dye, the (GO@Cu)-MB complex presented low degradation rate than GO and GO@Cu, which may be due to the masking action of the MB-dye, which delayed the pyrolysis action of the (GO@Cu)-MB complex.

#### 3.1.5. EDS Analysis

**Figure 4:**
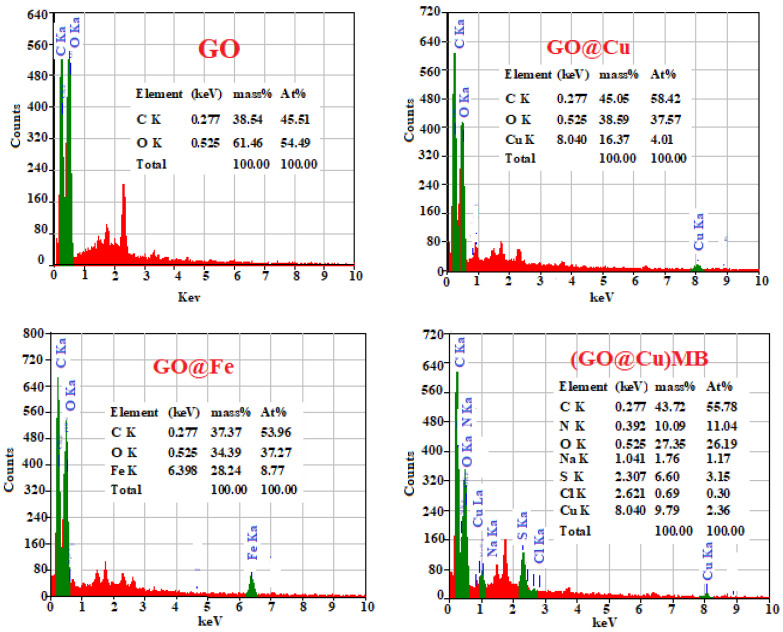

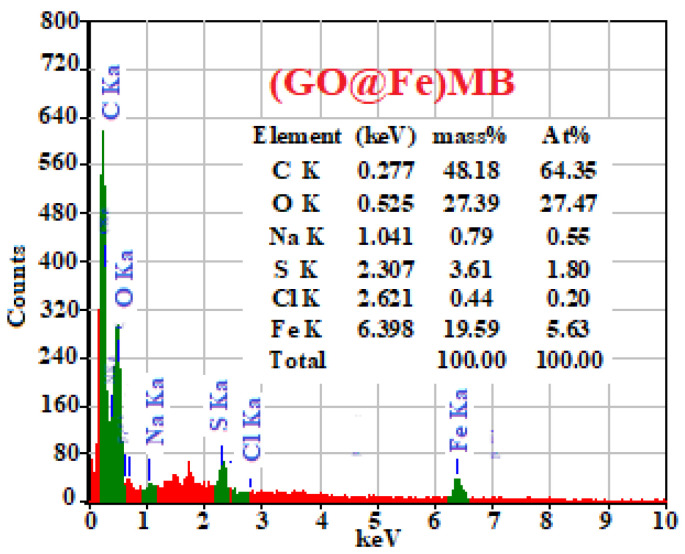
EDS analysis of GO@M and (GO@M)-MB, (M = Cu^2+^ or Fe^3+^).

The EDS analysis of the GO, GO@Cu, GO@Fe, (GO@Cu)-MB complex, and (GO@Fe)-MB complex were presented in Figure 4. The GO is a carbon material decorated with oxygen functional groups. Thus, it is mainly composed of C and O-atoms. The O-function groups was interacted with the M-ion by mixing the GO with the M-ion (Cu^2+^ or Fe^3+^). Therefore, the Cu^2+^ and Fe^3+^ were appeared in the EDS analysis of the GO@M, the MB-dye contains C, N, and S atoms. Thus, the EDS analysis results of the (GO@M)-MB involves N and S-atom as displayed in Figure 4.

### 3.2. Adsorption Parameters

#### 3.2.1. Effect of Contact Time

**Figure 5:**
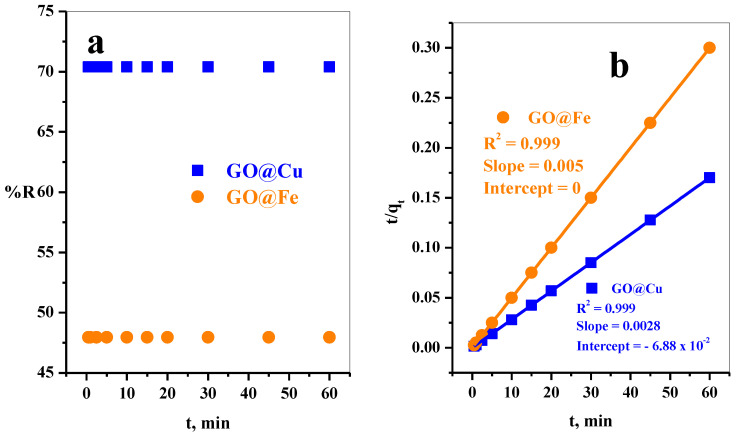
Effect of (**a**) contact time on removal percent, (**b**) Pseudo second-order plot of MB-dye adsorption using GO@Cu ([MB] = 50 mg L^−1^, Dose = 5 mg, V = 50 mL, pH = 7, T = 25 °C) and GO@Fe ([MB] = 50 mg L^−1^, Dose = 6 mg, V = 50 mL, pH = 7, T = 25 °C) from aqueous media.

The influence of stirring time on MB-dye adsorption on GO@Cu and GO@Fe adsorbents was displayed in Figure 5a. The dye adsorption from the aqueous media onto the two adsorbents in the same manner, where the equilibrium state was achieved spontaneously at the contact of the adsorbent with the aqueous dye solution. The highly rapid adsorption of the dye species over both adsorbents may be attributed to the flat structure and highly separated GO@Cu and GO@Fe adsorbent, reefer the TEM and SEM images (Figure 1). This arrangement will expose the most available active sites to the dye species, which will reduce the time required for interacting with the adsorbent with the MB-dye species.

##### Adsorption Kinetics Evaluation

Generally, the kinetic evaluation was set up by fitting the data obtained from the experiment to the pseudo-first-order and pseudo-second-order kinetic models. In this work, the equilibrium was achieved rapidly in the first 30 sec from contacting the GO@M and MB-dye. Therefore, the pseudo-first-order kinetic model will be excluded for adsorbent GO@Cu and GO@Fe.

The linear form of the pseudo-second-order model is represented as follows:

(2) $$\frac{t}{q_{t}\;}=\frac{1}{K_{2\;}q_{e}^{2}}+\frac{t}{q_{e}}$$

where q_e_ (mg g^−1^) is the amount of sorption at equilibrium time, q_t_ (mg g^−1^) is the amount of sorption at time t, and K_2_ (g mg^−1^ min^−1^) is the rate constant of the pseudo-second-order reaction.

(3) $$q_{e}=\frac{\left(C_{o}-C_{e}\right)V}{1000w}$$

where C_o_ is the initial concentrations (mg L^−1^), C_e_ is the MB-dye concentrations at equilibrium time intervals (mg L^−1^), V is the volume of MB-dye solution (mL), and w is the mass of GO@M (g).

(4) $$q_{t}=\frac{\left(C_{o}-C_{t}\right)V}{1000w}$$

where C_t_ is the MB-dye concentrations at various time intervals (mg L^−1^). 

By plotting t against t q_t_^−1^, Figure 5b, the obtained correlation coefficients, R^2^, and the analyzed values of slopes and the intercepts were summarized in Table 2. It was clear that the R^2^ = 0.999 for both adsorbents; moreover, the calculated adsorption capacities (q_ecal_, mg g^−1^) for GO-Cu (357.14) and GO-Fe (200.00) were highly closed to the experimental adsorption capacities (q_e exp_), refer Table 2. This explanation suggested that the adsorption kinetics of MB species over GO-Cu and GO-Fe can be described with a pseudo-second-order kinetic model.

#### 3.2.2. Effect of Initial Dye Concentration

**Figure 6:**
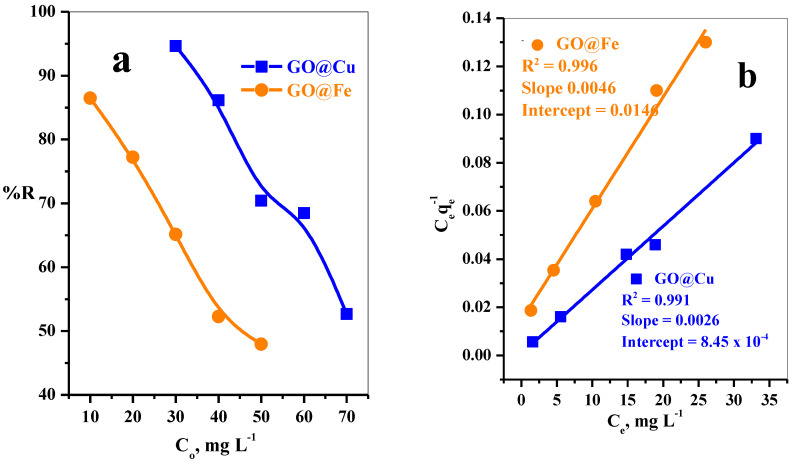

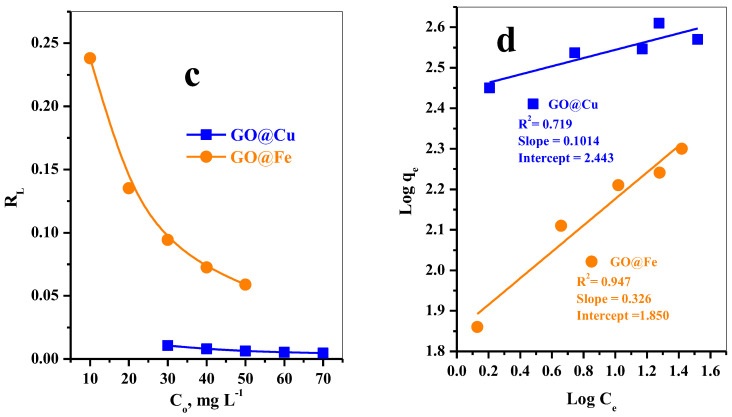
Effect of (**a**) dye concentration on removal percent, (**b**) Langmuir isotherm plot, (**c**) R_L_ plot and (**d**) Freundlich isotherm plot of MB-dye using GO@Cu (t = 5 min, Dose = 5 mg, V = 50 mL, pH = 7, T = 25 °C) and GO@Fe (t = 5 min, Dose = 6 mg, V = 50 mL, pH = 7, T = 25 °C) form aqueous solution.

Figure 6a illustrates the adsorption efficiency of MB-dye by both adsorbents GO@Cu and GO@Fe at MB-concentration range (30–70 mg L^−1^) and (10–50 mg L^−1^), respectively. Obviously, the further increase in the dye concentration will be followed by a retreating in its adsorption percent for both of the two adsorbents. As the MB-dye concentration increase, while the number of active sites is maintained constant, the ratio of the number of available active sites to the number of the dye molecules will tend to decrease; consequently, the removal percent will sever from the decrease. This behavior is attributed to, at low MB-dye concentrations, the number of available active sites is relatively large compared to the number of MB-dye molecules. Therefore, it will be recorded higher removal percent.

##### Adsorption Isotherm Study

The adsorption isotherm studies were investigated by fitting the adsorption data to Langmuir and Freundlich models. The mathematical forms of the two isotherm models are expressed by Equations (5) and (6), respectively.

(5) $$\frac{C_{e}}{q_{e}}=\left[\frac{1}{Q^{o}b}\right]+\left[\frac{1}{Q^{o}}\right]C_{e}$$

where q_e_ is the amount of MB-dye absorbed per unit weight of GO@M (mg g^−1^), C_e_ is the equilibrium concentration of MB-dye in the bulk of solution (mg L^−1^), Q^o^ is the monolayer adsorption capacity in mg g^−1^, and b is a constant related to the free energy of sorption processes. A plot of C_e_/q_e_ versus C_e_ gives a linear relation with Q^o^ and b calculated from the slopes and intercepts.

(6) $${\mathrm{logq}}_{e}{=\mathrm{logK}}_{f}+\frac{1}{n}{\mathrm{logC}}_{e}$$

where K_f_ in mg g^−1^ and n are the Freundlich constants related to sorption capacities and sorption intensities. 

The values of linear correlation coefficients (R^2^) resulting from Figure 6b,d, along with other parameters derived from the two isotherm models, are represented in Table 3. The analysis of the obtained data illustrated that the Langmuir model appears to fit well the adsorption data of both adsorbents (GO@Cu and GO@Fe), as expressed by the higher values related to R^2^ than those belonging to the Freundlich model as represented in Table 3. Such a trend explored the formation of monolayer adsorption of MB-dye species on the homogeneous energy active site’s surface. In addition, the maximum adsorption performance was 384.62 mg g^−1^ for GO@Cu and 217.39 mg g^−1^ for GO@Fe.

The feasibility of the process can be evaluated by a separation factor (dimensionless constant) “R_L_”, Figure 6c, which is given in the following Equation (7):

R_L_ = 1/(1 + bC_o_) (7)

The “R_L_” value located among zero and one for favorable adsorption, whereas unfavorable adsorption (R_L_ > 1), linear adsorption (R_L_ = 1), and irreversible adsorption (R_L_ = zero).

#### 3.2.3. Effect of Adsorbent Dose

**Figure 7:**
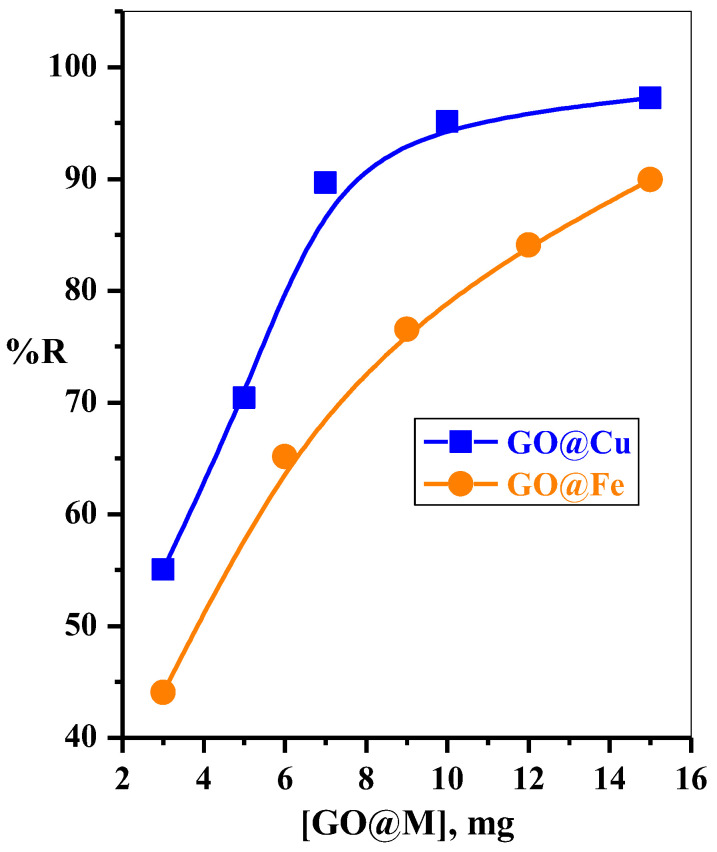
Effect of GO@M dose/50 mL dye solution on removal percent of MB-dye; GO@Cu (t = 2.5 min, [MB] = 50 mg L^−1^, V = 50 mL, pH = 7, T = 25 °C) and GO@Fe (t = 2.5 min, [MB] = 30 mg L^−1^, V = 50 mL, pH = 7, T = 30 °C) from aqueous solution.

The effects of GO@Cu and GO@Fe dosages on MB-dye remediation efficiency from aqueous solution were investigated in the range of 3–15 mg/50 mL and plotted in Figure 7. It was observed that the adsorption percentages of the MB-dye enhanced dramatically with an increase in the adsorbent dose (GO@Cu and GO@Fe) in the tested range. Such observation refers to increasing the density of the available active sites of both used adsorbents with further increasing adsorbent dosage.

#### 3.2.4. The Induce of pH and Adsorption Mechanism

**Figure 8:**
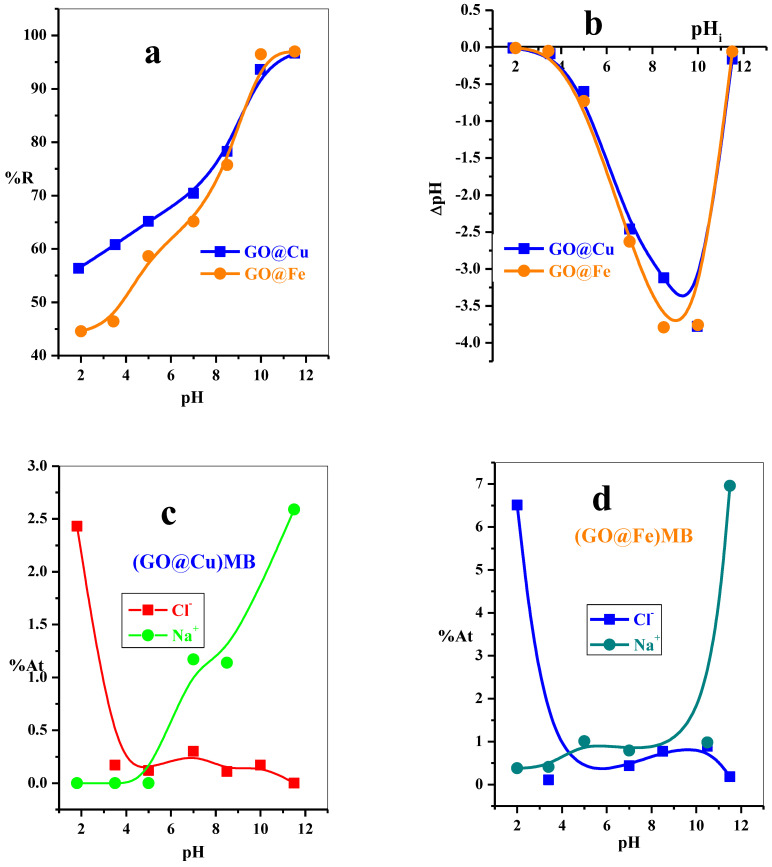
Effect of the initial solution pH on the (**a**) removal percentage (% R) from aqueous solution, (**b**) pH_ZPC_ of the GO-M, M = Cu^2+^ or Fe^3+^, adsorbent of MB-dye; GO-Cu (t = 2.5 min, [MB] = 50 mg L^−1^, dose = 5 mg, V = 50 mL, T = 25 °C) and GO-Fe (t = 2.5 min, [MB] = 30 mg L^−1^, dose = 6 mg, V = 50 mL, T = 25 °C) and (**c**,**d**) the %At of Cl^−^ and Na^+^ ions on the GO-M surface at different pH environment.

The pH parameter is regarded as an important parameter affecting the adsorption processes. The pH environment is induced by the ionization of both MB-species and the adsorbent surface functional groups. Herein, the effect of the pH values in range (1.9–11.5) on the adsorption performance of MB-dye using GO@Cu and GO@Fe was investigated, as displayed in Figure 8a. The adsorption efficiency of the MB species over both composites was detected to linearly increase with further increase in the pH values, as indicated in Figure 8a.

The pH values of the used dye solutions were determined before (pH_i_) and after (pH_f_), and the ΔpH (pH_f_ -pH_i_) were calculated. The point of zero net charges (PZC) was evaluated by plotting ΔpH against pH_i,_ as explored in Figure 8b. Interestingly, adsorbent GO@Cu and GO@Fe are negatively charged even at a low pH. Moreover, both composites’ negativity was increased as the solution pH values increased—consequently, the electrostatic attraction among the positively MB-species and the negatively adsorbent increased. As a result, a sharp increase in the adsorption percent with a further increase in the pH of the solution will be obtained, as illustrated in Figure 8b. 

The GO-sheet is completely loaded with the M-ion species. Moreover, the speciation of these M-ions alters according to pH ranges. Where, at low pH values, the M-ion will be presented in the cation form (M^z+^) with further increase in the pH, it will work for hydroxide complex M(OH)_2_; consequently, a higher hydroxide complex M(OH)_4_ will be formed at higher pH values. In this study, the electronegativity of the composites (GO@Cu and GO@Fe) were tracked in different pH environments (Figure 8b). At low pH values (1.9), the ΔpH ≈ 0 (i.e., the charges on the composite surface were neutral); therefore, the electrostatic attraction towards the cationic MB-dye will be weak adsorption percent is low. As the pH of the dye solution increases, the ΔpH value decreases (i.e., the density of the negative charges on the composites surface increases). This is attributed to the formation of GO@M(OH)_n_ complex, which will enhance the electrostatic attraction with the cationic MB-dye and the adsorption efficiency performed. In alkaline condition, the concentration of Na^+^ in the dye solution increased, which may be led to the formation of the GO@M(OH)(O-Na^+^) complex, which highly decreased the ΔpH value (at this condition, the surface of the composite were more negatively) which was favorable for excellent interaction with cationic dye species. Moreover, at a high alkaline solution (11.5), the ΔpH highly decreases; this may be attributed to the presence of large numbers of Na^+^ species in the dye solution, which will result in the accumulation of Na-ion on the surface of the composites and reduce its ΔpH, but the adsorption percent still high.

The EDS analysis (Figure S1) for the GO@M composite was set up after removing MB-dye from the aqueous solution at different pH conditions as shown in Figure 8c,d. The EDS analysis showed that the Cl^−^ ion present in the analyzed GO@M sample with a high concentration at pHi = 1.9 would reduce this concentration with further increased initial pH values. At low pH = 1.9, this is attributed to the composite surface carrying a neutral charge, GO-M^+^; hence, the Cl^−^ anions will be heaped upon the composite surface. The further increase in the pH will enhance the negative charge of the GO@M composite surface, which generates a repulsion force between the GO@M and Cl^−^ anions. On the contrary, the Na^+^ ion was not detected at pH_i_ < 7, after that, the %At of the Na^+^ in the GO@M sample highly improved. This behavior referred to, at pH_i_ > 7, the surface of the GO@M composite will be more negative and ionized in the presence of Na-ion; therefore, as the negativity of the GO@M increases, it will interact with a greater number of the Na-ions.

#### 3.2.5. The Effect of Sodium Chloride Dose

**Figure 9:**
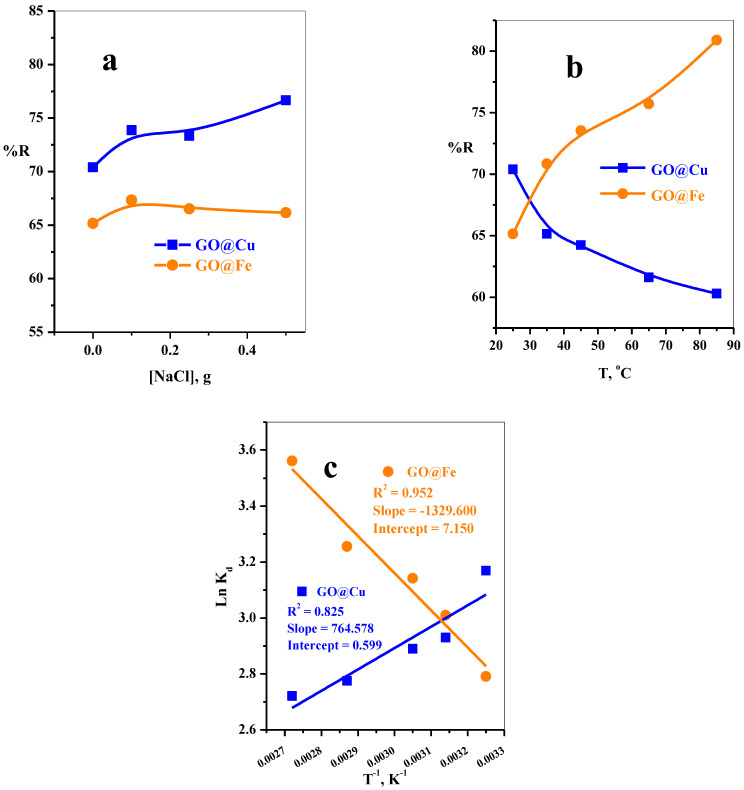
The effect of (**a**) sodium chloride dose, (**b**) solution temperature on the removal efficiency of MB using GO@Cu, and (**c**) Thermodynamic plots (t = 2.5 min, [MB] = 50 mg L^−1^, dose = 5 mg, V = 50 mL, T = 25 °C) and GO@Fe (t = 2.5 min, [MB] = 30 mg L^−1^, dose = 6 mg, V = 50 mL, T = 25 °C) from aqueous medium.

The sodium chloride dose parameter in the range (0–400 mg) was displayed in Figure 9a. The removal percent of MB molecules using GO@Cu adsorbent slowly increased from 70.40 to 76.66%, while quietly stable from 65.15 to 66.83% increase using GO@Fe. This behavior is provided that the salinity in the studied range has a moderated influence on the adsorption of MB-species over GO@M nanocomposites.

#### 3.2.6. The Effect of Aqueous Solution Temperature

The influences of the temperature of MB-dye solution in range (25–85 °C) on the adsorption of MB-species over the GO@Cu and GO@Fe complexes were investigated and illustrated in Figure 9b. The adsorption performance of MB-species of the GO@Fe complex was enhanced from 65.15 to 80.90% with a further increase in the studied temperature range. On the contrary, the GO@Cu possessed decreasing in the removal efficiency of the MB-species 70.40 to 60.30% when the temperature of the adsorption solution increased from 25 to 85 °C, respectively. Generally, the increase in the temperature of the dye solution will be continued with an increase in the mobility of the MB-dye molecules from the bulk of the solution to the GO-M nanocomposite surfaces; therefore, the adsorption efficiency will be increased, as in the case of GO@Fe composite. On the other hand, the decrease in the adsorption ability of the GO@Cu with a further increase in the solution temperature may be due to the increase in the temperature will result in decreased surface activity, hence, weakening of the physical bonds among the MB-species and the GO-M nanocomposite active sites, so the dye molecule tends to escape from the binding site of the adsorbent [19]. 

#### 3.2.7. Thermodynamic Analysis

The nature of adsorption of MB-species on the GO@Cu and GO@Fe adsorbents can be explained with the aid of thermodynamic parameters such as Gibbs free energy (ΔG^o^), enthalpy change (ΔH^o^), and entropy change (ΔS^o^), and these parameters could be evaluated by the following expressions (8) and (9):

∆G^o^ = −RT LnK_d_ (8)

where R is the universal gas constant (8.314 Jmol^−1^ K^−1^), and T is the temperature (K).

Ln K_d_ = ((∆S^o^)/R) − ((∆H^o^)/RT) (9)

By plotting Ln K_d_ vs. T^−1^, Figure 9c, and the parameters ΔS^o^ and ΔH^o^ can be calculated from the slopes and intercepts, respectively. Moreover, the values of ΔG^o^ were calculated from Equation 8. After that, the obtained values of ΔG^o^, ΔS^o^, and ΔH^o^ are summarized in Table 4. The values of ΔG^o^ were observed to be negative in the temperature range (308–368 K) for both adsorbents; this provided that the adsorption is a spontaneous process. Based on ΔH^o^, Table 4, the adsorption reaction of MB-species over the GO@Cu is exothermic and endothermic in the case of GO@Fe. Meanwhile, the positive values of ΔS^o^ explain the affinities of the adsorbents (GO@Cu and GO@Fe) for MB-dye, and the randomness increases at the interfaces of the solid solution while the adsorption process proceeds. 

#### 3.2.8. Selective Adsorption of Cationic Dyes 

**Figure 10:**
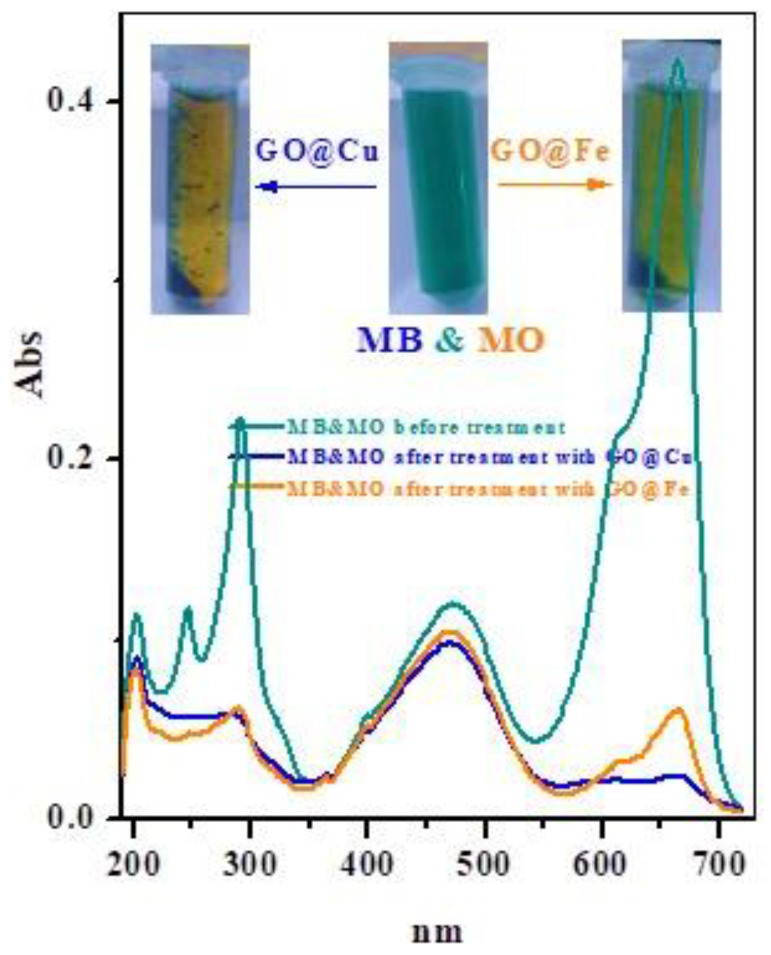
UV–vis spectra of (MB-MO) binary system solution before and after treated with GO@M (M = Cu^2+^ or Fe^3+^) composite. Inset: optical images that present the color of the MB-MO binary solution before and after being treated with GO@M.

Simulated waste adsorbent GO@Cu and GO@Fe ability to select adsorb the cationic MB-species from binary dye system of MB and MO-dye (MO-dye is anionic dye) had also been investigated, as presented in Figure 10. In this figure, the optical images of the MB&MO binary solution before and after the adsorption process using both adsorbents. The UV–vis spectrum of the binary solution presented peaks at (664 nm, MB) and (472 nm, MO); however, after contact with both adsorbents, the peak corresponding to MB-dye highly diminished the peak related to MO-dye was still present. These findings also prove electrostatic interaction among the MB-dye and GO@Cu and GO@Fe binding sites.

### 3.3. Comparison of MB-Maximum Adsorption Capacity with Various Adsorbents

Table 5 summarizes the maximum adsorption capacities of the different adsorbents newly published (2020–2022) applied for the adsorption of MB-dye from wastewater. In addition to economic cost and eco-friendly advantages, the comparative information provided that the GO@M adsorbents are highly effective in removing MB-dye and treating wastewater.

## 4. Conclusions

In this report, the fabricated nanocomposite succeeded in adsorption MB dye on the graphene oxide loaded with a heavy metal ion (Cu^2+^ and Fe^3+^). The used materials were characterized by different advanced techniques such as SEM, TEM, FTIR, Raman, and EDS before and after the adsorption of MB-dye. Attributed to the highly separated and flat structure (TEM analysis) of the obtained composites, the adsorption kinetics were highly enhanced. According to Langmuir, both composites possess excellent adsorption capacities compared with other adsorbents found in the literature. The thermodynamic properties showed the adsorption of MB-dye over GO@Cu exothermic and GO-@Fe endothermic reactions. Both composites show the additional property that can select the adsorption of cationic dyes.

## Figures and Tables

**Table 1:** The D and G-band positions, I_D_/I_G_ ratios, and FWHMs.

Sample	D-Band Peak		G-Band Peak		I_D_/I_G_
	Raman Shift (cm^−^^1^)	FWHM (cm^−^^1^)	Raman Shift (cm^−^^1^)	FWHM (cm^−^^1^)	
GO	1352	150	1598	112	1.34
GO-Cu	1339	119	1583	83	1.43
GO-Cu-MB	1339	119	1594	83	1.43
GO-Fe	1339	107	1582	72	1.49
GO-Fe-MB	1350	95	1605	83	1.14

**Table 2:** Pseudo-first-order and pseudo-second-order kinetic model parameters for adsorption of MB on GO@Cu ([MB] = 50 mg L^−1^, Dose = 5 mg, V = 50 mL, pH = 7, T = 25 °C) and GO@Fe ([MB] = 50 mg L^−1^, Dose = 6 mg, V = 50 mL, pH = 7, T = 25 °C) from aqueous media.

Dye	q_e exp_ (mg/g)	First-Order Kinetic Parameter			Second-Order Kinetic Parameter		
		K_1_ (min^−1^)	q_ecal_ (mg g^−1^)	R^2^	K_2_ (g mg^−1^ min^−1^)	q_ecal_ (mg g^−1^)	R^2^
GO-Cu	352.00				0.041	357.14	0.999
GO-Fe	199.83				0.00	200.00	0.999

**Table 3:** Langmuir and Freundlich constants for adsorption of MB-dye using GO@Cu (t = 5 min, Dose = 5 mg, V = 50 mL, pH = 7, T = 25 °C) and GO@Fe (t = 5 min, Dose = 6 mg, V = 50 mL, pH = 7, T = 25 °C) form aqueous solution.

Adsorbent	Langmuir Isotherm Model				Freundlich Isotherm Model		
	Q^o^ (mg g^−1^)	b (mg^−1^)	R_L_	R^2^	1/n	K_f_ (mg g^−1^)	R^2^
GO-Cu	384.62	3.10	0.011	0.991	0.1014	277.33	0.719
GO-Fe	217.39	0.32	0.238	0. 996	0.3260	70.80	0.947

**Table 4:** Standard thermodynamic parameters for MB-dye removal from aqueous solution utilizing GO@Cu (t = 2.5 min, [MB] = 50 mg L^−1^, dose = 5 mg, V = 50 mL, T = 25 °C) and GO@Fe (t = 2.5 min, [MB] = 30 mg L^−1^, dose = 6 mg, V = 50 mL, T = 25 °C) from aqueous medium.

T (K)	ΔG (kJmol^−1^)		ΔH (kJmol^−1^)		ΔS (Jmol^−1^ K^−1^)	
	GO@Cu	GO@Fe	GO@Cu	GO@Fe	GO@Cu	GO@Fe
308	−8.11	−7.15	−6.36	11.05	4.98	59.45
318	−7.75	−7.96				
328	−7.88	−8.57				
348	−8.03	−9.42				
368	−8.33	−10.90				

**Table 5:** Comparison of various adsorbents for removal of MB-dye.

Adsorbent	Adsorption Conditions				Q_o_, mg g^−1^	Ref.
	Adsorbent Dose (mg mL^−1^)	pH	Contact Time, Min	Dye Conc. (ppm)		
AF-U membrane	5	5	240	100	45.871	[20]
AF-S membrane	5	5	240	100	65.789	[20]
Humulus Japonicus Leaves (HJ)	15	7	20	100	145.77	[21]
Eucalyptus Camdulensis Biochar (Ec-bio)	5	6	60	50	123.30	[22]
calix[6]arene-modified PbS	44	6	60	20	5.495	[23]
Modified ZSM-5 zeolite	2	10	300	10	4.31	[24]
1-naphthyl ammonium tetrachloroferrate (III)	60 mg	3	120	40	9.52	[25]
pec/poly(MA-co-AMPS) hydrogel	0.5	7	60	200	448.40	[26]
CF	150	6.5	250	120	88.02	[27]
CF-CA	150	6.5	250	120	163.93	[27]
S. muticum	0.625	∗	120	10	157	[28]
mixed Sargassum	0.625	∗	120	10	115	[28]
PET-NFMWCNT	0.008 g	8	120	20	7.047	[29]
Alginate/PAA	0.05 g	8.5	90	10	120	[30]
FA-DMSN	10 mg	>7	3	50–125	90.7	[31]
Sargassum latifolium	0.1	10	15	40	0.819	[32]
*Vaccinium myrtillus* L. leaves powder	5	10	50	200	200.4	[33]
Modified Areca catechu Nut	2.8	≈12	180	300	333.3	[34]
Rt/BC	25 mg	8	∗	25–250	214.52	[35]
GO@Cu	0.1	7	2.5	50	384.62	This work
GO@Fe	0.12	7	2.5	30	217.39	This work

∗ Not detected.

## Data Availability

Data is contained within the article.

## Supplementary Materials

The following supporting information can be downloaded at: https://www.mdpi.com/article/10.3390/ma15103657/s1, Figure S1: EDS analysis of the GO-M after adsorption of MB at various pH.
