# Supplementary material for: Graphene Oxide@Heavy Metal Ions (GO@M) Complex Simulated Waste as an Efficient Adsorbent for Removal of Cationic Methylene Blue Dye from Contaminated Water
Source: Materials (Basel). 2022 May 20;15(10):3657. doi: 10.3390/ma15103657 (PMC9147086; doi:10.3390/ma15103657)
Supplement: Supplementary file 1 [file materials-15-03657-s001.zip › materials-1735387-SI.pdf]

materials

MDPI

Article

Graphene Oxide@Heavy Metal Ions (GO@M) Complex Simulated Waste as an Efficient Adsorbent for Removal of Cationic Methylene Blue Dye from Contaminated Water

Yangfan Ding [1], Zhe Chen [1], Jinglei Wu [1], Ahmed I. Abd-Elhamid [2], Hisham F. Aly [3], AbdElAziz A. Nayl [4,*] and Stefan Bräse [5,6,*]

1 Key Laboratory of Science and Technology, Eco-Textile & Shanghai Engineering Research Center of Nano-Biomaterials and Regenerative Medicine, College of Chemistry, Chemical Engineering and Bio-Technology, Donghua University, Shanghai 201620, China; dingyangfan@mail.dhu.edu.cn (Y.D.); chenzhe@mail.dhu.edu.cn (Z.C.); jw@dhu.edu.cn (J.W.)
2 Composites and Nanostructured Materials Research Department, Advanced Technology and New Materials Research Institute, City of Scientific Research and Technological Applications (SRTA-City), New Borg Al-Arab 21934, Egypt; ahm_ch_ibr@yahoo.com
3 Hot Laboratories Center, Egyptian Atomic Energy Authority, Cairo 13759, Egypt; alydrhisham@yahoo.com
4 Department of Chemistry, College of Science, Jouf University, Sakaka 72341, Al Jouf, Saudi Arabia
5 Institute of Organic Chemistry (IOC), Karlsruhe Institute of Technology (KIT), Fritz-Haber-Weg 6, 76133 Karlsruhe, Germany
6 Institute of Biological and Chemical Systems-Functional Molecular Systems (IBCS-FMS), Director Hermann-von-Helmholtz-Platz 1, 76344 Eggenstein-Leopoldshafen, Germany
* Correspondence: aanayel@ju.edu.sa or aanayl@yahoo.com (A.A.N.); stefan.braese@kit.edu (S.B.)

Citation: Ding, Y.; Chen, Z.; Wu, J.; Abd-Elhamid, A.I.; Aly, H.F.; Nayl, A.A.; Bräse, S. Graphene oxide@heavy Metal Ions (GO@M) Complex Simulated Waste as an Efficient Adsorbent for Removal of Cationic Methylene Blue Dye from Contaminated Water. *Materials* **2022**, *15*, 3657. https://doi.org/10.3390/ma15103657 ma15103657

Academic Editor: Sabino De Gisi

Received: 5 May 2022
Accepted: 18 May 2022
Published: 20 May 2022

Publisher's Note: MDPI stays neutral with regard to jurisdictional claims in published maps and institutional affiliations.

Copyright: © 2022 by the authors. Licensee MDPI, Basel, Switzerland. This article is an open access article distributed under the terms and conditions of the Creative Commons Attribution (CC BY) license (https://creativecommons.org/licenses/by/4.0/).

Abstract: Graphene oxide (GO) was heavily used in the adsorption process of various heavy metal ions (such as copper (Cu) and iron (Fe) ions), resulting in a huge waste quantity of graphene oxide@metal ions complex. In this research, the authors try to solve this issue. Herein, the GO surface was loaded with divalent (Cu^{2+}) and trivalent (Fe^{3+}) heavy metal ions as a simulated waste of the heavy metal in various removal processes to form GO@Cu and (GO@Fe) composites, respectively. After that, the previous nanocomposites were used to remove cationic methylene blue (MB) dye. The prepared composites were characterized with a scanning electron microscope (SEM), transition electron microscope (TEM), Fourier transmission infrared (FTIR), Raman, and energy-dispersive X-ray (EDS) before and after the adsorption process. Various adsorption factors of the two composites towards MB-dye were investigated. Based on the adsorption isotherm information, the adsorption process of MB-dye is highly fitted with the Langmuir model with maximum capacities (mg g^{-1}) (384.62, GO@Cu) and (217.39, GO@Fe). According to the thermodynamic analysis, the adsorption reaction of MB-species over the GO@Cu is exothermic and, in the case of GO@Fe, is endothermic. Moreover, the two composites presented excellent selectivity of adsorption of the MB-dye from the MB/MO mixture

Keywords: graphene oxide; heavy metal ions; nanocomposite; cationic dye; adsorption; water treatment

S1. Experiments

S1.1. Materials and Instrumentation

All chemicals were of analytical grade and used as received. Sulfuric acid (95–97%, Riedel deHaen), hydrogen peroxide (30%, Pharaohs Trading and Import), hydrochloric acid (36%, El Salam for Chemical Industries), potassium permanganate (99%, Longlive), and graphite (200 mesh, 99.99%, Alpha Aesar) Methylene Blue (MB) (Sigma-Aldrich), Sodium Nitrite ($NaNO_2$, Curlo Erba) Phosphoric acid (Pharaonic Company for Trade and

Import), Copper nitrate trihydrate (Himedia Laboratory Pvt Ltd, Extra pure), Ferric chloride anhydrous (Fisher Scientific UK Limited, 99%) and Methyl Orang (MO) (Sigma-Aldrich). The main equipment used are the following; pH meter (3510, Genway), hot plate stirrer (SB 162, Stuart, UK.), Centrifuge, (Mikro 220R, Hettich, UK.), Analytical balance (CP 2245, Sartorius, USA.), and (UV/Vis. Spectrophotometer-Double beam (T80+, PG instruments Ltd., UK.).

S1.2. Characterization

Scanning electron microscope model: (SEM, JEOL GSM-6610LV) and transition electron microscope model: (TEM, JEOL GSM-6610LV, Japan) were used for investigating the surface morphology of resultant composite. Fourier transmission infrared spectroscopy (FT-IR) model: (8400s Shimadzu, Japan) with a wavelength range from 4000 to 400 cm^{-1}. Raman spectra were provided using Raman Microscope (Bruker, SenterraII, Germany) with an excitation wavelength of 514 nm and a power of 5 mW. Thermogravimetric analyzer model: (Shimadzu thermal gravimetric analysis (TGA)—50, Japan) was investigated to detect the thermal stability of resultant composites. Thermal decomposition of samples was carried out under nitrogen with a heat rate of 10 °C/min.

S2. Results and Discussion

More details show in Figure S1 below.

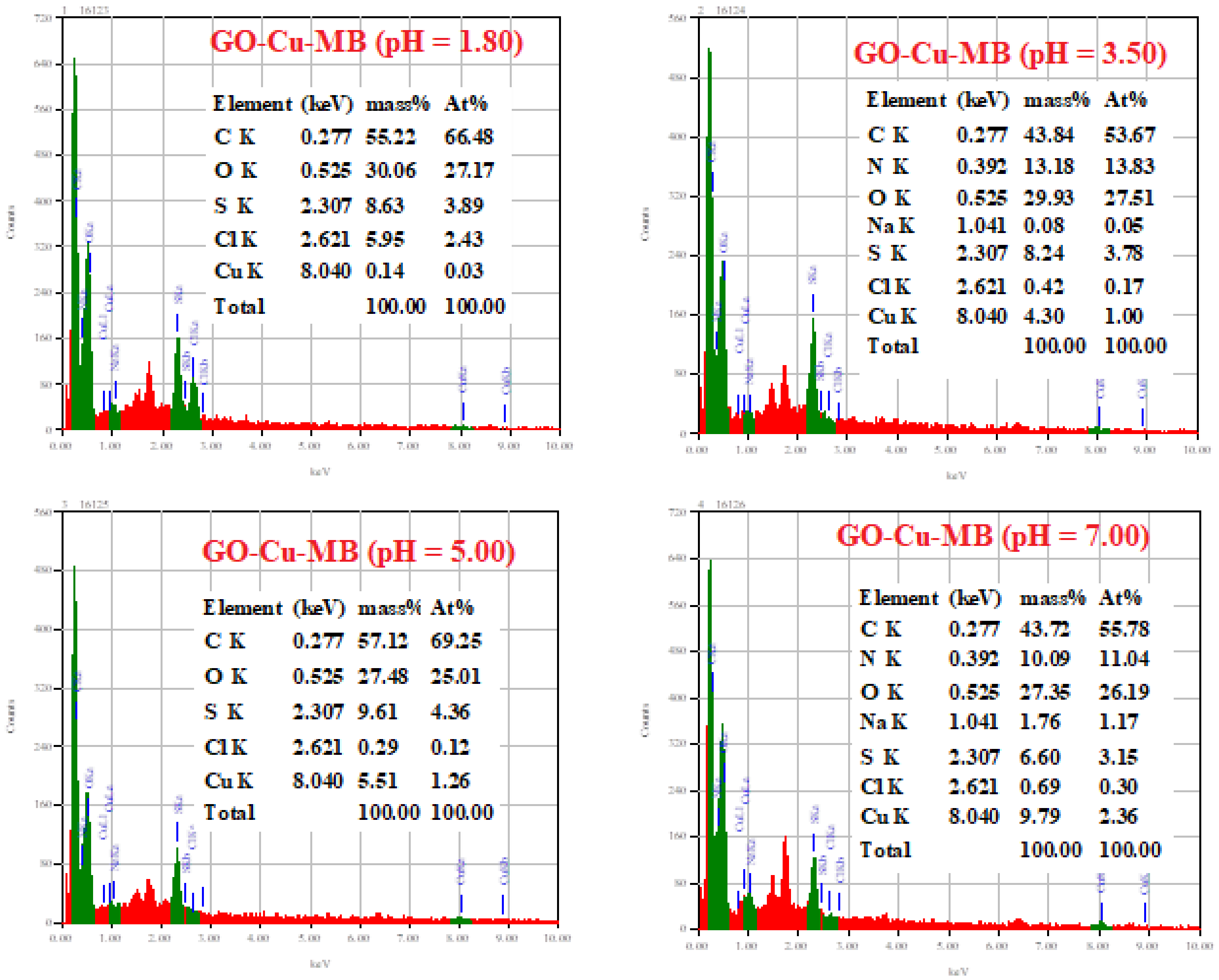

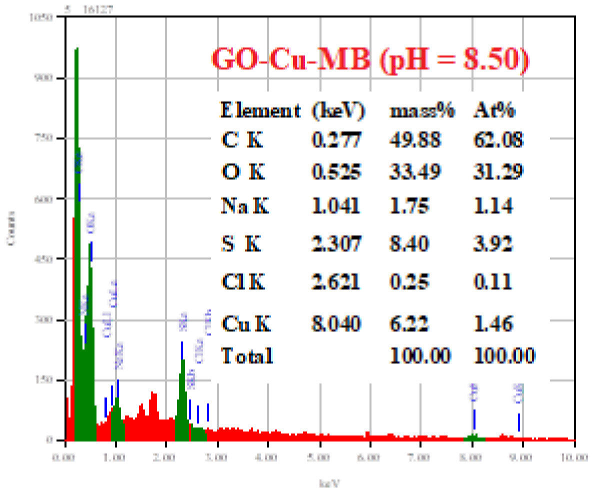
GO-Cu-MB (pH = 8.50)
Element (keV) mass% At%
C K 0.277 49.88 62.08
O K 0.525 33.49 31.29
Na K 1.041 1.75 1.14
S K 2.307 8.40 3.92
Cl K 2.621 0.25 0.11
Cu K 8.040 6.22 1.46
Total 100.00 100.00

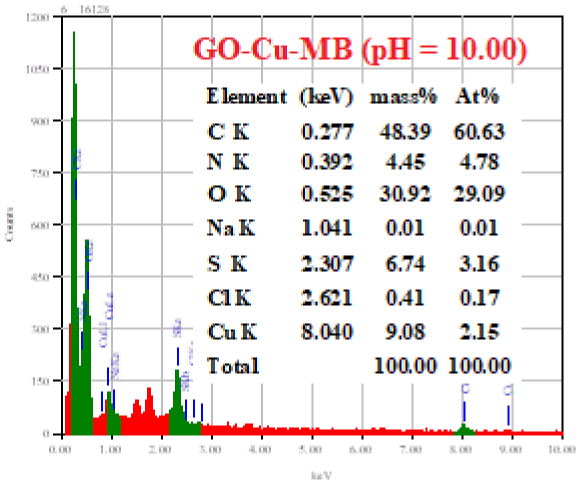
GO-Cu-MB (pH = 10.00)
Element (keV) mass% At%
C K 0.277 48.39 60.63
N K 0.392 4.45 4.78
O K 0.525 30.92 29.09
Na K 1.041 0.01 0.01
S K 2.307 6.74 3.16
Cl K 2.621 0.41 0.17
Cu K 8.040 9.08 2.15
Total 100.00 100.00

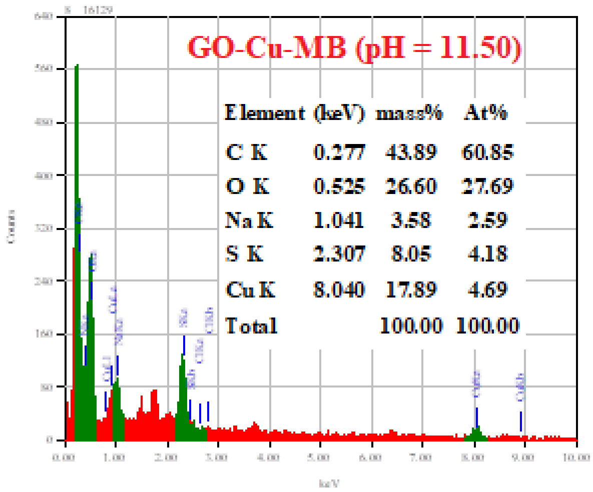
GO-Cu-MB (pH = 11.50)
Element (keV) mass% At%
C K 0.277 43.89 60.85
O K 0.525 26.60 27.69
Na K 1.041 3.58 2.59
S K 2.307 8.05 4.18
Cu K 8.040 17.89 4.69
Total 100.00 100.00

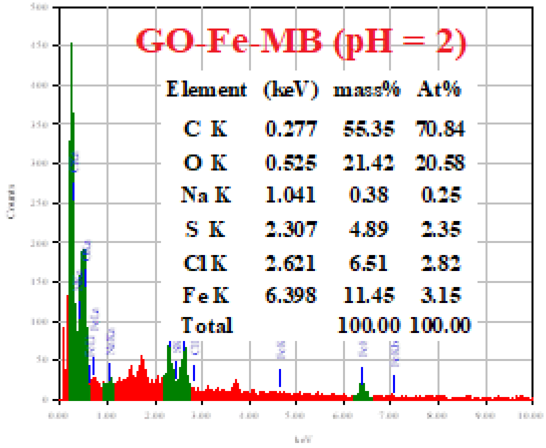
GO-Fe-MB (pH = 2)
Element (keV) mass% At%
C K 0.277 55.35 70.84
O K 0.525 21.42 20.58
Na K 1.041 0.38 0.25
S K 2.307 4.89 2.35
Cl K 2.621 6.51 2.82
Fe K 6.398 11.45 3.15
Total 100.00 100.00

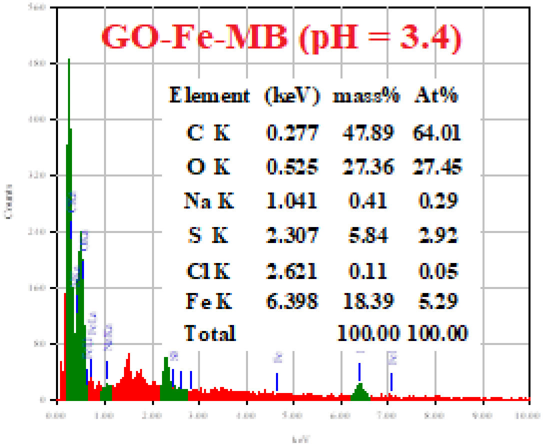
GO-Fe-MB (pH = 3.4)
Element (keV) mass% At%
C K 0.277 47.89 64.01
O K 0.525 27.36 27.45
Na K 1.041 0.41 0.29
S K 2.307 5.84 2.92
Cl K 2.621 0.11 0.05
Fe K 6.398 18.39 5.29
Total 100.00 100.00

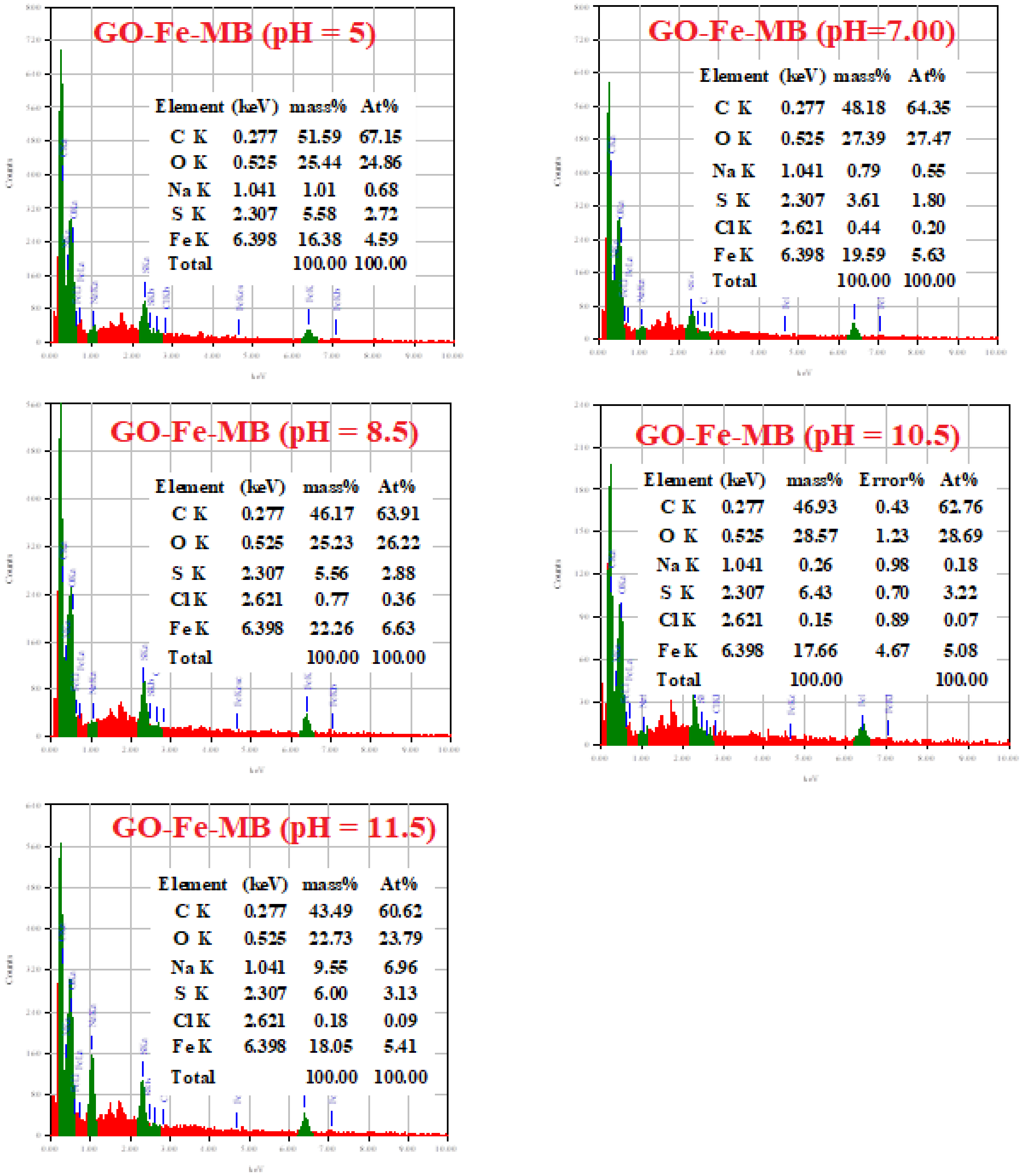

Figure S1. EDS analysis of the GO-M after adsorption of MB at various pH.
